# Endoscopic surgery: One picture worth thousand words

**Published:** 2017-06

**Authors:** Arnoud Wattiez

**Affiliations:** Prof OBGYN University of Strasbourg, France; Head of GYN Department, Latifa Hospital, Dubai- UAE

When the camera was first connected to the endoscope in 1984, we did not imagine the undergoing revolution yet! At that time the pessimists claimed that nothing could be better than the human eye. But plugging this camera not only enhanced the quality of vision, improved the surgeon’s comfort and the ergonomic, but also allowed to share the vision to all the actors of the surgery. Above this and most important, it opened the way to a new method of understanding and teaching surgery. Beyond the signal delivered by the camera and restituted to the screen it is not only what is seen, it is what is understood. That’s why this “apparent” simple evolution has turned out to become a revolution and allowed endoscopic surgery to develop, to improve and to be teached and reproduced in safe conditions for the patient.

Having a constant electronic signal not only allows us to see and teach directly, it can also be stored and kept for comparison fulfilling the real mean of medicine which is documentation. What kind of medical report could better compare situations at different times and the effect of a surgical treatment? What can better give you the exact pre-operative and post- operative patient’s status that can lead to a correct study of the outcomes?

It can be useful to check a possible missed adverse effect that could explain an abnormal clinical evolution. It can be stored to constitute a library that represents the experience of the surgeon and its evolution. It can be a witness of what has been done to the patient: right or wrong. It’s also the only way to store surgical procedures allowing the beginner to view them again and again until no steps are unknown.

That’s why a video corner open for endoscopic procedures becomes mandatory in peer-reviewed scientific journals. It will deal with interesting and challenging clinical cases, surgical techniques and significant original situations but also will present didactic cases useful to train youngsters and trainees.

Doing a video of a surgical case is not easy. It requires from the surgeon an analysis of what has been done and a selection of the right moment to be shown. Again the pessimists will claim that those videos do not represent the truth! That’s right, but they will represent the ideal situation that every surgeon aims to reach!

Needless to say that all contributions have to be peer-reviewed following strict guidelines.

We invite all endoscopists to publish in the journal. No matter the location, the settings, the affiliation or curriculum vitae. From wherever in the world the journal will be the opportunity to share experience and receive the expertise of a great number of surgeons.

